# Ionmob: a Python package for prediction of peptide collisional cross-section values

**DOI:** 10.1093/bioinformatics/btad486

**Published:** 2023-08-04

**Authors:** David Teschner, David Gomez-Zepeda, Arthur Declercq, Mateusz K Łącki, Seymen Avci, Konstantin Bob, Ute Distler, Thomas Michna, Lennart Martens, Stefan Tenzer, Andreas Hildebrandt

**Affiliations:** Institute of Computer Science, Johannes Gutenberg University, 55128 Mainz, Germany; Institute for Immunology, University Medical Center of the Johannes Gutenberg University, 55128 Mainz, Germany; Immunoproteomics Unit, Helmholtz-Institute for Translational Oncology (HI-TRON), 55131 Mainz, Germany; VIB-UGent Center for Medical Biotechnology, VIB, 9052 Gent, Belgium; Department of Biomolecular Medicine, Ghent University, 9000 Ghent, Belgium; Institute for Immunology, University Medical Center of the Johannes Gutenberg University, 55128 Mainz, Germany; Institute of Computer Science, Johannes Gutenberg University, 55128 Mainz, Germany; Institute of Computer Science, Johannes Gutenberg University, 55128 Mainz, Germany; Institute for Immunology, University Medical Center of the Johannes Gutenberg University, 55128 Mainz, Germany; Institute for Immunology, University Medical Center of the Johannes Gutenberg University, 55128 Mainz, Germany; Immunoproteomics Unit, Helmholtz-Institute for Translational Oncology (HI-TRON), 55131 Mainz, Germany; VIB-UGent Center for Medical Biotechnology, VIB, 9052 Gent, Belgium; Department of Biomolecular Medicine, Ghent University, 9000 Ghent, Belgium; Institute for Immunology, University Medical Center of the Johannes Gutenberg University, 55128 Mainz, Germany; Immunoproteomics Unit, Helmholtz-Institute for Translational Oncology (HI-TRON), 55131 Mainz, Germany; Institute of Computer Science, Johannes Gutenberg University, 55128 Mainz, Germany

## Abstract

**Motivation:**

Including ion mobility separation (IMS) into mass spectrometry proteomics experiments is useful to improve coverage and throughput. Many IMS devices enable linking experimentally derived mobility of an ion to its collisional cross-section (CCS), a highly reproducible physicochemical property dependent on the ion’s mass, charge and conformation in the gas phase. Thus, known peptide ion mobilities can be used to tailor acquisition methods or to refine database search results. The large space of potential peptide sequences, driven also by posttranslational modifications of amino acids, motivates an *in silico* predictor for peptide CCS. Recent studies explored the general performance of varying machine-learning techniques, however, the workflow engineering part was of secondary importance. For the sake of applicability, such a tool should be generic, data driven, and offer the possibility to be easily adapted to individual workflows for experimental design and data processing.

**Results:**

We created ionmob, a Python-based framework for data preparation, training, and prediction of collisional cross-section values of peptides. It is easily customizable and includes a set of pretrained, ready-to-use models and preprocessing routines for training and inference. Using a set of ≈21 000 unique phosphorylated peptides and ≈17 000 MHC ligand sequences and charge state pairs, we expand upon the space of peptides that can be integrated into CCS prediction. Lastly, we investigate the applicability of *in silico* predicted CCS to increase confidence in identified peptides by applying methods of re-scoring and demonstrate that predicted CCS values complement existing predictors for that task.

**Availability and implementation:**

The Python package is available at github: https://github.com/theGreatHerrLebert/ionmob.

## 1 Introduction

Ion mobility enhanced mass spectrometry coupled with liquid chromatography (LC-IMS-MS) improves throughput and coverage of proteomics experiments (Meier *et al.* 2015). Traditionally, proteomics analyses have been performed by separation of peptides using reversed phase liquid chromatography (LC), interfaced by electrospray ionization to mass spectrometry (MS) to analyze the mass to charge ratio (*m*/*z*) of the analyte ions. Ion mobility separation (IMS) adds an additional dimension of separation. Its functionality is based on the fact that in the presence of an electric field, small, compact ions will behave differently when flying through a cloud of charge-neutral gas than larger ones (Valentine *et al.* 2005). This enables to distinguish molecules, such as isobaric ions with the same *m*/*z* but differing sequences or modifications, that can’t be separated by LC-MS (Meier *et al.* 2021b, Ogata *et al.* 2021). Additionally, IMS allows for filtering or untargeted annotation (Dodds and Baker 2019).

Depending on the hardware setup, the IMS can be used to calculate the collisional cross-section (CCS) value of the ions by applying kinetic theory that links experimentally determined reduced ion-mobilities to their momentum transfer collision integral through the Mason–Schamp equation (Revercomb and Mason 1975, Gabelica *et al.* 2019). The translation of the reduced mobility, *K*0, to CCS depends on several factors including ion mass and charge, as well as the mass and temperature of the drift gas. However, the application of this theory makes some simplifying assumptions including that the electric field applied is low enough to be negligible. As a result, the translation of experimentally determined drift times to CCS can only be performed for low field devices such as drift tube, travelling wave, or trapped ion mobility (DTIMS, TWIMS, and TIMS), but not for devices involving high fields such as field asymmetric ion mobility spectrometry (Dodds and Baker 2019). For an in-depth description of differences in the determination of *K*0 from experimental setups see e.g. Gabelica and Marklund (2018), Dodds and Baker (2019), and Gabelica *et al.* (2019). As the ion CCS is an inherent physicochemical property, CCS calculations from IMS data are highly reproducible (Bush *et al.* 2012, Meier *et al.* 2021a). Thus, LC-IMS-MS offers both increased capability of ion separation and identification crucial for the ultimate task of elucidating the chemical composition of samples.

There are two principal reasons for establishing a CCS predictor. First, it offers potential insight into how specific features can influence the ion conformation in the gas phase (Ogata *et al.* 2021, Meier *et al.* 2021a). These could be used to drive particular experimental setups tailored to certain types of peptides (Gomez‐Zepeda *et al.* 2023). Second, given their high reproducibility, CCS of ions hold valuable information ready to be integrated into candidate scoring. Thus, it can help to increase the confidence and overall number of identifications of peptides. Especially the second point is of particular importance, as in bottom-up proteomics one has to also perform the peptide to protein inference. Both factors can improve the coverage of peptides and thus of proteins identified.

Recently, there has been steadily increasing interest in the prediction of peptide CCS values (Meier *et al.* 2021a, Chang *et al.* 2021, Samukhina *et al.* 2021, Zeng *et al.* 2022). This task boils down to designing a function defined over some space of arguments resulting in a (real-valued) CCS prediction, which can be easily recognized as a regression task. In contrast to a simple database look-up, the key advantage of this approach comes from the possibility to obtain predictions for previously unobserved data. For example, a given sample might result in ions with sequences that were previously not observed. A prominent example is posttranslational modifications (PTMs) derived from biological conditions (e.g., phosphorylation) or experimental setups (e.g., carbamidomethylation). IMS can help to differentiate peptides with identical sequences but different PTM positions, which may have different biological results. Another relevant example is MHC ligands sensed by the immune system to trigger the defense against possible threats, such as cancerogenic cells or virus infections. Detecting MHC peptide ligands, also called immunopeptides, is essential for developing vaccines and immunotherapies. MHC ligands result from the cleavage of proteins mediated by diverse enzymes in the cell, exponentially expanding the search space and thus complicating their identification. In addition, immunopeptidomics samples are more likely to contain isobaric peptides than proteomics samples due to conserved patterns in their sequences. Since LC-MS alone cannot separate such peptides, IMS becomes essential to improve the identification of MHC ligands. Overall, the space of observable ions is huge, but there exists complex yet stable principles that govern the observed CCS values.

To establish such a predictor, several modeling strategies are available. Historically, due to small number of available reference data-points, mostly low-parametric approaches were used. One of the first was based on intrinsic size parameters (ISPs; Henderson *et al.* 1999), where every amino acid is assigned some fixed value *a priori*. ISPs were derived from a set of 660 peptide sequences. A prediction of CCS for a specific sequence is then carried out by simply summing over all contributions. Applicability for enhanced peptide identification based on ISPs has also been discussed (Valentine *et al.* 2011). This idea was later extended to also account for e.g. PTMs by adding up contributions of individual atoms instead of amino acids (Kaszycki and Shvartsburg 2017). Other approaches included a multi-layer perceptron architecture and support vector machines (Wang *et al.* 2009, 2013) that relied on engineered features derived from 595 peptide sequences. While those models are quick to run and already do offer a lot of insight into the problem, they simply cannot fully account for the richness of the configuration space of ions.

With the increased use of LC-IMS-MS methods into mainstream proteomics and the resulting increase in the availability of measured CCS values (Meier *et al.* 2021b), more complex data-driven approaches were proposed. Using positional encoded ISPs with a linear regression model was implemented by a very recent study by Chang *et al.* (2021). This way, increased expressiveness of the predictor was achieved with features that could be extracted from a training dataset of 135 000 peptide sequences. A very different approach was taken by Meier *et al.* (2021a), where the authors trained a deep recurrent neural network end-to-end. This was possible due to the generation of a training set with ≈550 000 examples. Their model achieves state-of-the art prediction accuracy on a test set of ≈150 000 unique test sequences. Samukhina *et al.* (2021) also built upon this dataset using deep learning. They applied a mixed architecture of 1-D convolutions and handcrafted features for sequences in combination with model-averaging, which resulted in increased prediction accuracy. To give a complete picture of potential approaches, reasoning based on physics were also performed, where one used Monte Carlo molecular dynamics to simulate the CCS values (de Carvalho *et al.* 2013, Kondalaji *et al.* 2017, Villatoro *et al.* 2019). However, these simulations are prohibitively demanding in terms of computational time and therefore impractical to use in high-throughput scenarios.

Overall, there are many similarities in the techniques used currently to model ion mobilities to those used for retention times (Ma *et al.* 2018, Gessulat *et al.* 2019), i.e. the LC-derived measurements. Given the much higher stability of the ion mobility measurements, it is interesting to ask what these models can bring to the table.

Apart from the proper choice of the architecture of the predictor, additional steps have to be typically performed to preprocess the data. From the practical standpoint, these steps take most of the actual work of the data-scientist. These steps include solving issues such as outlier detection, deduplication, feature generation and alignment of new data. We present here ionmob—a Python package for preprocessing datasets and fitting predictors of the CCS values of peptides identified by LC-IMS-MS using data acquired in timsTOF instruments. It offers routines necessary to solve all of the practicalities mentioned above and results in a prediction accuracy matching the state-of-the-art models. In addition, we included a selection of pretrained models and architecture primitives, which are easy to integrate into existing workflows. Importantly, ionmob integrates trypsin-cleaved peptides, phosphorylated tryptic peptides and also MHC ligands into deep learning driven CCS prediction, making our models more expressive. The package is well documented and available free of charge under the MIT license from github. Finally, we investigated how *in silico* prediction of CCS could be utilized to improve identification of peptides, allowing to integrate additional information from LC-IMS-MS proteomics experiments. ionmob will facilitate the training and incorporation of CCS-prediction in diverse workflows for LC-IMS-MS proteomics and peptidomics experiments.

## 2 Approach

### 2.1 General ionmob workflow

**Figure 1.**
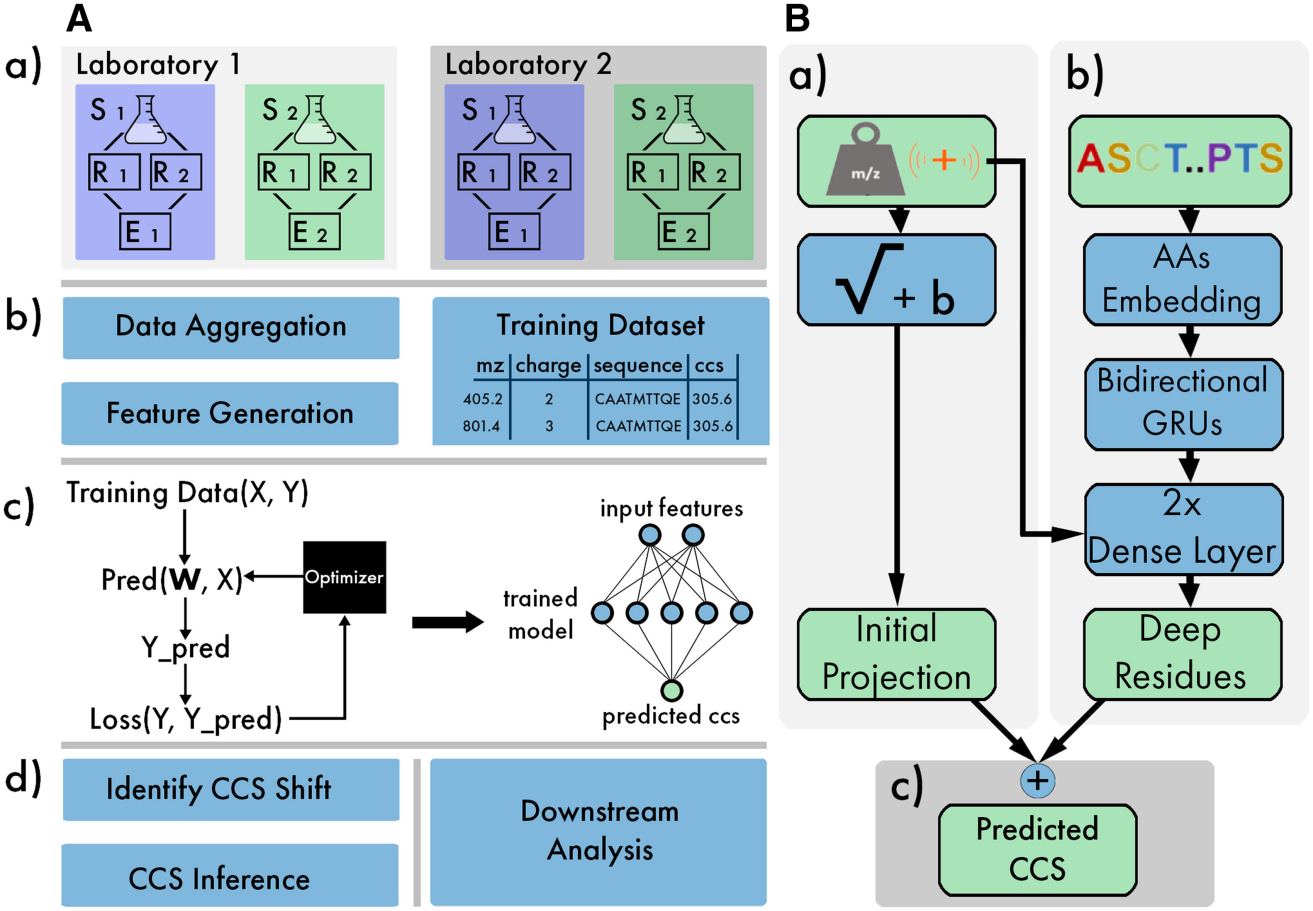
(A) General workflow of ionmob. (a) Data are generated from different samples, devices and laboratories. A sample of interest ($S_{1}$, $S_{2}$) is analyzed through multiple replicates ($R_{1}$, $R_{2}$) and combined into an identification table during raw data analysis ($E_{1}$, $E_{2}$). (b) For a representative set of training values, peptide charge state pairs are pre-processed e.g. deduplicated. Raw data are then translated into sets of features for machine learning. This results in data ready for training. (c) Training then is an iterative process where the internal state of a predictor is changed so that its output better resembles the desired output based on some objective measure. This results in a trained model that can be used for prediction. (d) Before trained model outputs can be compared with data derived from a new source, a dataset specific shift needs to be calculated. After that, predictions of a model are ready, e.g. for rescoring. (B) Proposed model architecture. (a) Simple initial projection fitting a square-root function and a bias with mass and charge of a peptide as inputs. (b) Recurrent neural network using GRUs to predict higher-order interactions that contribute to observed CCS based on peptide sequences. Deeper dense layers are also provided with the charge state of the ion as additional input. AAs stands for amino acids. (c) Final CCS values are then calculated as sum of initial projection and deep residues

An ionmob workflow can be composed of one or more building blocks, depending on the task at hand. An overview is given in Fig. 1A.

The regular occurring tasks roughly fall into three categories: data preprocessing, model training, and CCS inference. We provide explicit functionality for:

- detection of peptides with multiple conformations,
- deduplication of data points followed by an aggregation strategy,
- feature generation, e.g. tokenization, based on data from multiple software sources,
- alignment of new datasets for optimal prediction performance, and
- pre-trained models for CCS inference out-of-the-box.

### 2.2 Modeling strategy and predictor architecture

**Figure 2.**
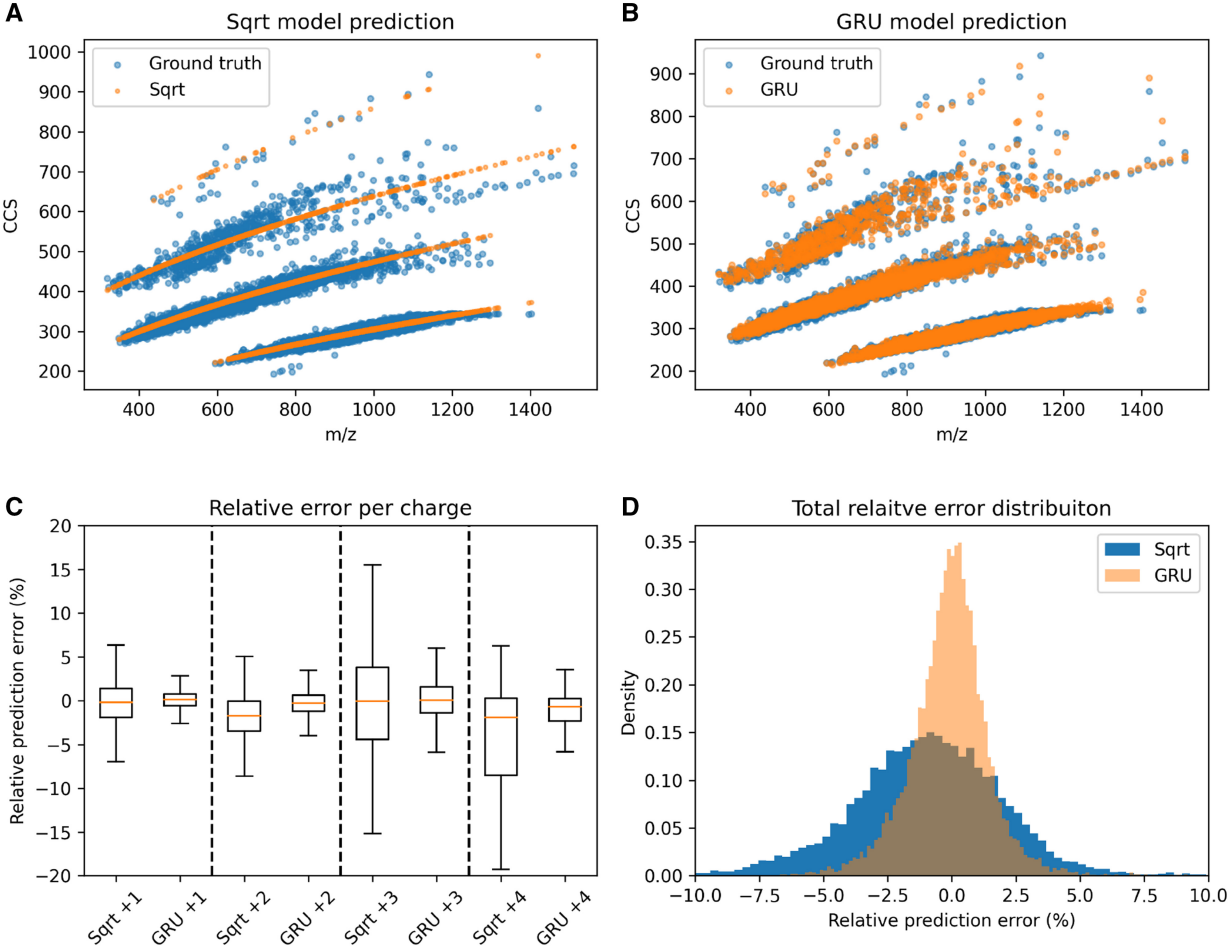
*m*/*z* versus CCS for observed (blue) and predicted (orange) CCS values of MHC peptides, model performance. (A) Ground truth versus predicted CCS after initial projection with a simple square-root function, see Equation (1) and Fig. 1Ba. (B) Final CCS prediction as sum of initial projection and deep residues, see Equation (2) and Fig. 1Bc. (C) Boxplots showing charge state wise relative errors comparing both prediction accuracies. (D) Total relative error distributions for both models after training

Previous models used machine-learning algorithms to predict CCS directly from peptide sequence and charge. However, the CCS of an ion is highly correlated with its mass and charge (Meier *et al.* 2021b) (see Fig. 2). This fact can be exploited to reformulate the prediction task to only predict the residues with respect to an initial projection of mass and charge.

The reformulation yields multiple advantages: first, it reduces the convergence time of training considerably since the starting initial CCS is already much closer to the real value. Second, fitting a regression model with gradient-based optimization was numerically unstable for models with few parameters. This could be because the output domain of a model needs to span a wide range of values and higher charge states are underrepresented compared with lower ones. Third, having this simple component of the predictor separated from more complex ones makes it convenient to look at the contributions of higher-order interactions of features. Fourth, it also establishes a baseline accuracy (see Fig. 2).

We therefore decided to rephrase our approach. First, an initial CCS value is calculated solely based on an ions mass and charge, see Equation (1), where a coefficient $w_{c}$ and an intercept $b_{c}$ are fit separately for each modeled charge state *c a priori*.

(1) $${\text{CCS}}_{\text{init}}(\text{mz},c)=w_{c}\sqrt{\text{mz}}+b_{c}$$

Afterward, a regressor *M* with parameter set θ is fit to further lower the mean absolute error (MAE) of predicted CCS values compared with the experimentally observed ones, see Equation (2).

(2) $${\text{CCS}}_{\text{final}}(\text{mz},c,s|M)={\text{CCS}}_{\text{init}}(\text{mz},c)+M(s,c,\theta )$$

Input features may vary for different implementations of *M* but are all based on information derived from peptide sequence *s* and charge state *c*. A concrete implementation is shown schematically in Fig. 1B, representing our proposed predictor architecture. It has ≈550 000 trainable weights.

Here, the residues with respect to the initial square-root fit are modeled by a deep recurrent neural network, using token embeddings for amino acids and bidirectional gated recurrent units (GRUs; Chung *et al.* 2014) to provide a sequence specific contribution (see Fig. 2).

## 3 Materials and methods

### 3.1 Sample preparation and LC-IMS-MS data acquisition

The detailed methodology for sample preparation and LC-IMS-MS analysis is included in Supplementary Material and Methods. LC-IMS-MS was performed in nanoAcquity (Waters) or nanoElute (Bruker) chromatography systems connected to either timsTOF Pro-2 or timsTOF SCP (Brunner *et al.* 2022) (Bruker) MS, using DDA-PASEF (Meier *et al.* 2018) for MS acquisition.

### 3.2 Hardware, raw data analysis, and regression modeling

MaxQuant version 2.0.3.0 (Cox and Mann 2008, Sinitcyn *et al.* 2021) and PEAKS XPro version 10.6 (BSI, Canada) were used to process generated .d raw files. Results used for training were filtered at 1% FDR at the peptide-spectrum match (PSM) and peptide level. For phosphopeptides, only identifications with a PTM AScore > 20 were used (99% confidence). Data preprocessing, model training and package development were performed on a workstation running Ubuntu 20.04 with 32 GB of RAM, an AMD Ryzen 7 3700X 8-Core Processor and a NVIDIA RTX 2070 SUPER GPU with CUDA version 11.2 and cuDNN 8.1.1. Peptide identification was performed with the andromeda search engine and reference X-FASTA sequences. All regression models were implemented with scikit-learn (Pedregosa *et al.* 2011) version 1.1.0, scipy (Virtanen *et al.* 2020), version 1.8.0 or TensorFlow (Abadi *et al.* 2016), and version 2.9.0 using Python 3.9.

### 3.3 Data aggregation

The peptide identification text files created by MaxQuant and PEAKS were used to generate our in-house datasets. Feature duplicates consisting of (sequence, charge, and CCS) instances were aggregated and their occurrences tracked. In order to distinguish between differently folded populations of peptides with the same charge state and sequence but notably different CCS values, modality classes were assigned. Features with identical charge and sequence that diverge the boundary of 2-times the standard deviation to the main feature are considered to be secondary features. Thereby, a distinction between unimodal and bimodal distributions of CCS values for a feature is established. Within the bimodal fraction of features, a main feature is determined based on the peptide mode with highest occurrence. Features that displayed CCS values within the boundary of 2-times the standard deviation to the main feature are considered as part of the main feature and therefore aggregated/fused. The difference in CCS values for the latter features are attributed to measurement inaccuracies. The resulting CCS value is the occurrence-weighted average of all (sequence, charge)-duplicates within the same modality class. In case of multi-modality of a feature, only the main feature was kept.

### 3.4 External datasets

We downloaded the peptide identification tables from the following publicly available datasets acquired in timsTOF instruments.

1. The tryptic peptide training and test sets published by Meier *et al.* (2021a) (data preparation described therein) available at github from their repository (https://github.com/theislab/DeepCollisionalCrossSection, accessed 10.07.22). The combined dataset contained 718 917 unique pairs of charge state and sequence.
2. The tryptic peptide results published by Chang *et al.* (2021) that were made accessible through jPOST, dataset id JPST 000959, JPST 001017, and JPST 001176. The dataset was deduplicated by the same strategy described for our own dataset, see previous section. Sequence charge state pairs that were already present in one of the other datasets were removed, which left a total of 5064 examples.
3. The results published by Feola *et al.* (2022) (MHC ligands) and Ogata *et al.* (2021) (phosphopeptides) that were made accessible through PRIDE, dataset id PXD 026463 and jPOST, dataset id PXD 019746. The datasets were deduplicated by the same strategy described for our own dataset, see previous section. Sequence charge state pairs that were already present in one of the other datasets were removed, which left a total of 7366 examples and 7742 examples, respectively.

We extracted 42 sequence, charge state pairs acquired using $N_{2}$ as drift gas and measured twice, once with TWIMS and once with DTIMS from Bush *et al.* (2012) for evaluation of CCS prediction performance on non-timsTOF data.

### 3.5 Alignment of collisional cross-section values

A dataset specific, linear shift in CCS was calculated by matching sequence and charge state pairs identified in both the dataset obtained from Meier *et al.* and each of the other datasets (in-house and external). Using this dataset as reference is practical, as it holds by far the most sequences. This aligns the means of all observed CCS values in different datasets and is necessary to avoid systematic error.

### 3.6 Training, validation, and test set generation

A concatenation of training and test sets published by Meier *et al.* (2021a) together with our in-house generated phosphorylation and MHC ligand datasets were used as training set, the in-house generated tryptic dataset was used as validation set. The three remaining external datasets were later used as test sets.

### 3.7 Model training

Initial square-root fit was performed separately for each charge state using SciPy, see Equation (1). Resulting parameter values were then used to parameterize a custom tensorflow layer with nontrainable weights. Model optimization was performed with gradient descent using MAE as objective function. The Adam optimizer was used with an initial learning rate of $10^{-3}$. Dropout regularization with a dropout rate of 0.2 was applied between the last two deep dense layers. After each epoch, MAE was calculated on the validation set. If there was no decreased MAE for at least three epochs, the learning rate was lowered by an order of magnitude. If there was still no improvement on the validation set for another three epochs, training was stopped.

### 3.8 External collisional cross-section predictors

We downloaded the CCS predictor tools described by Meier *et al.* (2021a), Zeng *et al.* (2022), and Samukhina *et al.* (2021). We ran those models on our three test datasets according to the provided instructions. In the latter case, we therefore calculated the resulting CCS value for a given sequence and charge state pair as the average of all five created predictions.

### 3.9 Calculation of additive scalar amino-acid properties

The pepdata Python package (v1.0.7; https://github.com/openvax/pepdata) was used to calculate scalar features for peptide sequences. The package provides scalar descriptors of different amino-acid properties like hydropathy or polarity as mappings from a given amino acid to the respective value. For the scalar descriptors volume, polarity, hydropathy, hydrophilicity, solvent-exposed area, accessible surface area, accessible surface area folded, local flexibility, and pK side-chain, we calculated their normalized value by summing over the individual contributions of amino acids per sequence and divided by the sequence length.

### 3.10 Rescoring with collisional cross-section features

We tested the impact of using CCS predictions by adding CCS features as feature set to the current implementation of MS²Rescore (v2.1.2; Declercq *et al.* 2022) in addition to the existing peak intensity and retention time prediction features. For this, the mzid PEAKS search engine output files from the tryptic, phosphorylated and MHC ligand peptide data were used as is. Furthermore the MHC peptide dataset was acquired using a method optimized to include singly charged peptides (Gomez‐Zepeda *et al.* 2023). All three were parsed with the PEAKS pipeline of MS²Rescore; however, the pin files were modified with additional CCS features before running percolator. These features include the observed and predicted CCS value, the error, the absolute error and the percentual CCS error between observed value and predicted value. Peptides that carried >4 charges were left out since the CCS predictor was not trained for these charges. Subsequently, these modified pin files were rescored with percolator (The *et al.* 2016) (v3.05.0) as well as a pin files without these features to be able to compare the effects of CCS features when rescoring. The additional rescoring analyses were done in jupyter notebooks and plots were generated using matplotlib(v5.3.2) and seaborn(v0.11.2).

## 4 Discussion

### 4.1 Model accuracy and comparison

**Figure 3.**
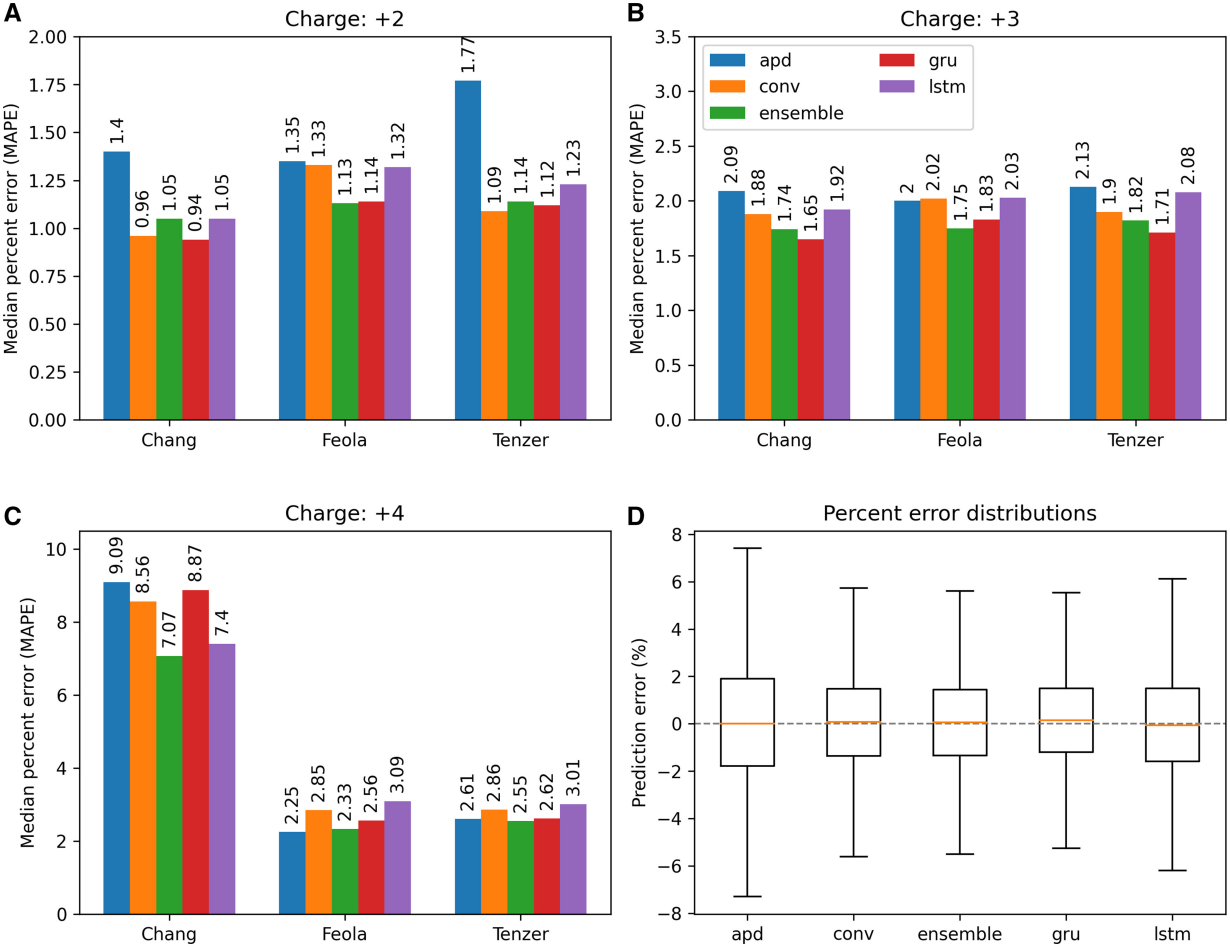
A performance comparison between ionmob GRU predictor and freely available deep predictors. (A–C) Performance per charge state for different test datasets. The gru model has a slight performance boost over the others for the Feola *et al.* (2022) dataset, likely since in contrast to the others it was explicitly trained on MHC peptides. Surprisingly, for charge state 4, prediction error for the Chang *et al.* (2021) dataset is relatively high for all models. (D) Boxplots of relative error distributions for all models. Overall performance of conv Samukhina *et al.* (2021), lstm Meier *et al.* (2021a), and gru model are relatively on par with each other, while the apd Zeng *et al.* (2022) model seems to perform a little worse. The ensemble prediction is calculated as the average predicted CCS value over all four models

To evaluate the performance of our model based on bidirectional gated recurrent units (gru), we compared it with three previously published deep predictors: Meier *et al.* (2021a) (lstm) using long-short term memory cells, Samukhina *et al.* (2021) (conv) using a mix of handcrafted features, convolutions, and model averaging, Zeng *et al.* (2022) (apd) using transformer-style attention. The results are shown in Fig. 3. Median absolute percent error (MAPE) was used as comparison metric, since the spread of CCS values for a given charge state increases with the mass of the ion (see Fig. 2).

Other studies predicting CCS made use of the Pearson correlation coefficient as an additional metric. However, we observed that the square-root baseline already had a correlation value of ≈0.97 and therefore think that it adds no significant insight into model performance. Interestingly, it can be observed that both the long-short term memory (lstm) and convolutional model (conv) show performances comparable to our predictor (gru) on the dataset from Chang *et al.* (2021) and our in-house tryptic dataset. However, they show a lower performance on the dataset from Feola *et al.* (2022). This could stem from the fact that the latter dataset contains MHC ligands, a type of peptide not present in the dataset those models were trained on but part of our in-house generated dataset. The CCS and IMS patterns of MHC ligands may be different since their C-ter amino acid is not necessarily Arg or Lys, as it is usually the case for tryptic peptides analyzed in proteomics experiments (Purcell *et al.* 2019). It hints that even if the authors of Meier *et al.* (2021a) could not observe significant improvements in prediction accuracy for their model beyond a training set size of ≈300 000 examples, the richness of naturally occurring peptides and experimental conditions might not be fully explored yet. Achieved accuracy on phosphorylated peptides could only be evaluated for our predictor and the model from Zeng *et al.* (2022) and is shown in Supplementary Fig. S6.

### 4.2 Prediction of collisional cross-section for singly charged peptide ions

In most proteomics workflows, the MS is configured to skip the fragmentation of singly charged ions or they are simply not considered for peptide identification during data processing (Prianichnikov *et al.* 2020, Gomez‐Zepeda *et al.* 2023, Purcell *et al.* 2019). This is because, first, singly charged peptides are more difficult to identify since only one of the two fragments is charged and can be detected after collision-induced dissociation. Second, most contaminants are singly charged as well and can be ignored thereby (Declercq *et al.* 2022). This also means that the range of inverse ion-mobility, where experimental measurements can be reliably transferred from reduced mobilities to CCS, will rarely cover those ions, since the instrument resolution is finite and tuned to the regions of highest interest (see Supplementary Fig. S7). This changes for MHC ligand peptides, as those can be singly charged due to their nontryptic origin, hence missing the C-terminal arginine or lysine as well as being relatively short (Declercq *et al.* 2022). We acquired an additional dataset of MHC peptides using Thunder-DDA-PASEF, which is explicitly set-up to cover those ions (Gomez‐Zepeda *et al.* 2023).

### 4.3 Comparing predicted collisional cross-section to experimentally acquired values from different ion mobility separation devices

As mentioned above, CCS values are never directly measured but derived from experimentally determined ion-mobilities by e.g. the Mason–Schamp equation or using a calibration curve (Dodds and Baker 2019). However, it is still a matter of ongoing debate under which experimental conditions this holds true (Gabelica and Marklund 2018). To gain insight whether or not our predictor might be applicable for IMS acquisitions that do not use TIMS separation, we compared predicted CCS values with results published by Bush *et al.* (2012). Here, a custom Synapt MS (Waters Corp.) with both a traveling-wave IMS (TWIMS) and a drift tube IMS (DTIMS) devices was used to derive CCS values for a total of 42 peptide sequence, charge state pairs that had 27, 11, and 4 sequences of charge state 2, 3, and 4, respectively. Results are graphically shown in Supplementary Fig. S5. The prediction accuracy was highest in the TWIMS analyses for ions with charge two and three (MAPE of 0.81 and 1.78, respectively) and for doubly charged ions in the DTIMS analyses (1.06). The error increased for ions with charge four in TWIMS (1.85) and ions with charge three or four in DTIMS (2.48 and 3.3, respectively). While these results offer preliminary insights, it is important to note that they were based on a very small sample. Therefore, gathering more data for evaluation would be necessary to confirm these findings. However, at the time of publication, we did not find available published data including the CCS for a larger number of peptides from any of these devices. In summary, although larger datasets are required to validate these results, ionmob has the potential to be applied to predict IMS in other instruments.

### 4.4 Driving factors of collisional cross-section prediction

#### 4.4.1 Embeddings of amino acids

**Figure 4.**
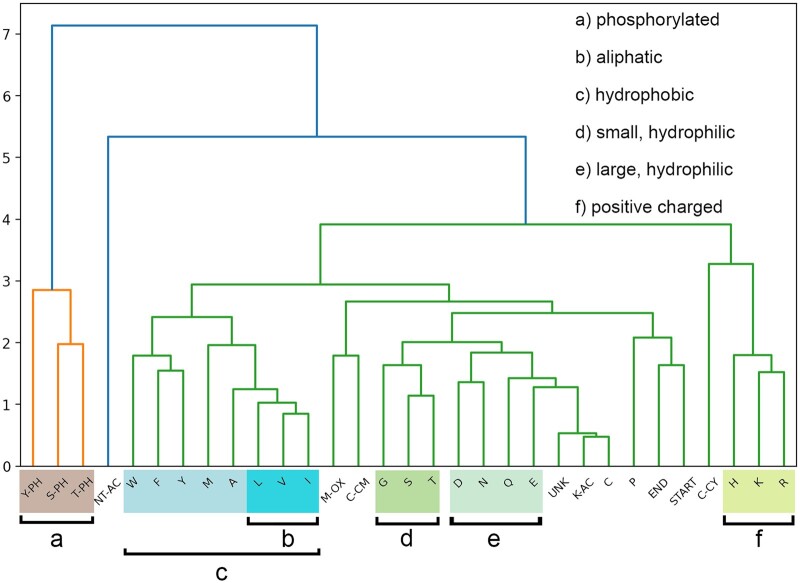
Agglomerative clustering of amino acid and modification embedding vectors. Outgroups are formed by phosphorylated, acetylated and positively charged amino acids. Inner groups are roughly divided between aliphatic and aromatic as well as hydrophilic and hydrophobic amino acids

The learned residues of peptide CCS values with respect to the initial fit use an embedding (here, 128 dimensional) to represent amino acids as dense vectors before being fed to the GRU units. This gives the opportunity to inspect how unmodified and modified amino acids are grouped relatively to each other in the embedding vector space by the network. To explore it, we evaluated the agglomerative clustering of amino acid features as shown in Fig. 4. Outgroups are formed by the phosphorylated amino acids Y, S, and T, acetylated N-termini and lastly the three positively charged amino acids H, K, R, and cysteinylated C. The inner clusters roughly fall into aliphatic or aromatic as well as hydrophilic or hydrophobic groups. Even though the network was not presented with any chemical or physical descriptors of the individual amino acids, information about them is learned from the relationship between sequences, charges, and the resulting CCS values.

#### 4.4.2 Correlation of deep residues with additive scalar descriptors of peptide sequences

Besides evaluating the individual relationships between amino acids in the embedding space, we also explored the linear relationships between sequence-wise predicted deep residues and additive scalar properties that can be directly calculated from amino acid sequences. A recent study, Chang *et al.* (2021) identified that increased hydropathy contributed to a higher CCS values, which we could observe as well. Additionally, we observed that local flexibility increases the overall relative CCS value of a given sequence as well. Those correlations are only moderate (0.55 and 0.53, respectively) but are in congruence with the relative grouping of individual amino acids in the embedding space of the predictor (see Fig. 4). All calculated Pearson correlations are visualized in Supplementary Fig. S4.

#### 4.4.3 Impact of phosphorylation on collisional cross-section predictions

In a recent study, Ogata *et al.* (2021) confirmed on a large scale that phosphorylation of peptides often leads to a more compact configuration of the ion in the gas phase, thereby lowering observed CCS values for modified peptide sequences compared with the unmodified version of these sequences. This effect outweighs the increase in peptide mass induced by such a modification (Ogata *et al.* 2021). Our analyses reproduced this finding for our in-house generated datasets and, consequently, this effect can also be observed when looking at synthetic predictions. We evaluated this by analyzing predicted CCS values for synthetically phosphorylated sequences with one phosphorylation site added at random compared with the unmodified sequence. We calculated the pairwise median relative percent decrease in predicted CCS of synthetically modified peptides compared with unmodified peptides per charge, which were 2.08, 2.85, and 3.86 for the charge states 2, 3, and 4, respectively (see Supplementary Fig. S1). The integration of *in-silico* predicted CCS for phosphorylated peptides into identification workflows might therefore be an excellent opportunity to be specifically integrated, which is already under commercial development (www.bruker.com/ru/news-and-events/news/2022/bruker-releases-ccs-enabled-timscore.html, accessed: 12.07.2022).

In summary, the GRU model implemented in ionmob not only enables the prediction of peptide CCS values from tryptic, nontryptic and modified peptides but also allows the evaluation of feature-specific contributions at the amino acid, sequence, and PTM levels.

### 4.5 Raw-data mobility distributions

**Figure 5.**
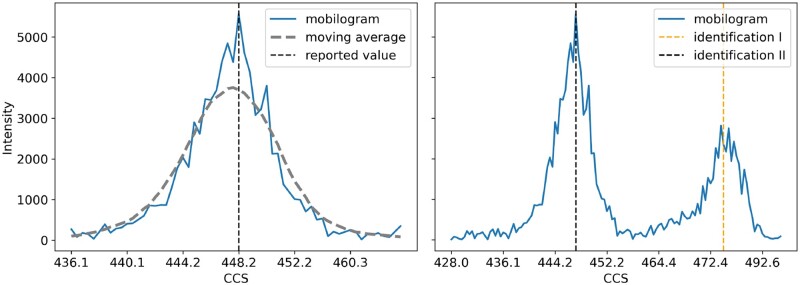
Marginal distributions of intensity along the ion-mobility dimension of peptide features, recorded with a timsTOF instrument. Left: Intensity distribution along the scan dimension (blue) for a uni-modal peptide feature, reported CCS is calculated from apex value (black). Right: intensity distribution for a multi-modal peptide. MaxQuant reported this peptide twice at the same retention time with differing scan indices (orange, red). Raw data extracted using opentimsŁącki *et al.* (2021). 1/K0 was converted to CCS using the Mason–Schamp equation

To establish prediction tools, one highly relevant question is whether or not the ion-mobility signal distribution should be taken into account when predicting CCS values. So far, all approaches including our own were implemented as maximum likelihood estimators. The simplification allows for a more straightforward formulation of the mobility modeling task. However, following this approach might be disadvantageous if the underlying assumption that signal distribution displays a single maximum does not hold. Notably, we and others observed that single peptide sequences can sometimes have multiple conformations in the gas-phase (Meier *et al.* 2021a). This in turn then results in a multi-modal distribution of ion mobility and an observable split of the ion clouds for charge state 3 and 4 (see Fig. 5).

Similar to the approach described by Meier *et al.* (2021a), we dealt with multi-modality of peptide collisional cross sections by first identifying and then removing all occurrences except the one with highest abundance. For a maximum likelihood estimation strategy, which will otherwise converge to their mean value, this is a necessary step. This strategy can be justified by the overall low number of multi-modal peptides, here ≈3%–5%. However, it obviously removes potentially valuable information and one should therefore try to derive driving factors of multi-modality. If identified, they could in turn be used to decide for a given prediction instance *a priori* if it might be necessary to predict more than one CCS value. Due to the relatively low number of candidates and potential stochastic processes driving multi-modality, we consider this to be an open challenge for future investigations (Table 1).

**Table 1.** Number of PSMs and unique identified peptides in terms of sequence for 1% for rescoring with or without CCS features.

Dataset	PSMs	unique peptides	CCS features
Tryptic	60 850	2901	+
	60 850	2882	−
Phospho	224 109	29 046	+
	223 595	28 928	−
MHC ligands	392 092	20 232	+
	389 113	20 130	−

### 4.6 Impact of collisional cross-section features in peptide-spectrum match rescoring

Using CCS predictions as an additional feature set in rescoring of database search results can provide increased performance to further separate true from false targets and gain confidence in the identified PSMs. To evaluate the value of CCS prediction for identification rescoring, we incorporated the ionmob models to the features used by MS^2^Rescore (Declercq *et al.* 2022) for the evaluation of true positive identifications. Rescoring including CCS features shows similar results for all three datasets, tryptic, phosphorylated, and MHC ligands (including singly charged), with a small increase in total number of identified PSMs and peptides, with the biggest increases seen for the MHC ligand dataset (Table 1). When investigating this dataset more closely, we found that 38.4% of the PSMs identified with CCS features that were not identified without these features are singly charged. Furthermore, all of these had a very low percentual CCS error, in contrast to singly charged PSMs that previously were identified and now not anymore due to the CCS features, where a lot of outliers are seen (Supplementary Fig. S2). Moreover, this trend was also seen in the other datasets where peptidoforms that were removed in comparison with rescoring without CCS features had higher errors. Even though the total number of identifications does not increase spectacularly, the feature weights show that they are indeed used by percolator when rescoring PSMs (Chang and Lin 2008). It demonstrates the value of using these features when rescoring PSMs, especially for the immunopeptidomics dataset (Supplementary Fig. S3). CCS features offer valuable information alongside peak intensity and retention time features allowing the rescore algorithm to select the best features for separating true from false targets for each database specifically. This filtering capability can be highly valuable for immunopeptidomics, where a high certainty level of identification is required to select the best targets for vaccines and immunotherapies.

## 5 Conclusion

We implemented ionmob, a framework for preprocessing, training and inferring peptide CCS values. ionmob includes a novel network architecture that combines a simple function fit with gated recurrent units, helping to stabilize and speed up training and make model outputs easier to interpret. By including phosphorylated peptides and MHC ligands also covering singly charged ions into the training set, we extend the applicability of our model, enabling the accurate prediction of a wider variety of PTMs than recently published models. Furthermore, our results suggest that local flexibility of peptides is an additional driving factor of increased CCS and confirm recently published findings that phosphorylation lowers CCS, which could be indicative of charge interaction-based compaction in the gas phase. Lastly, we demonstrate that *in silico* predicted CCS values can be used to increase confidence in peptide identifications by applying methods of rescoring. In summary, ionmob is an easily deployable package for the training and incorporation of peptide CCS prediction.

## Supplementary Material

btad486_Supplementary_Data

## Data Availability

All generated training, validation, and test datasets as well as all notebooks created during the experiments presented in this study have been deposited and made publicly available via Zenodo: https://zenodo.org/record/7516255. The mass spectrometry immunopeptidomics data have been deposited to the ProteomeXchange Consortium (Vizcaíno *et al.* 2014) via the jPOSTrepo partner repository (Okuda *et al.* 2017) with the dataset identifiers PXD043026 for ProteomeXchange and JPST002158 for jPOSTrepo.
