# Supplementary material for: Ionmob: a Python package for prediction of peptide collisional cross-section values
Source: Bioinformatics. 2023 Aug 4;39(9):btad486. doi: 10.1093/bioinformatics/btad486 (PMC10521631; doi:10.1093/bioinformatics/btad486)
Supplement: btad486_Supplementary_Data [file btad486_supplementary_data.pdf]

picture(0,0)(-35,0)(1,0)30 (0,35)(0,-1)30 picture picture(0,0)(35,0)(-1,0)30 (0,35)(0,-1)30 picture

Bioinformatics
doi.10.1093/bioinformatics/xxxxxx
Advance Access Publication Date: Day Month Year
Original Paper

OXFORD

Data and Text Mining

Ionmob: A Python Package for Prediction of Peptide Collisional Cross-Section Values

David Teschner [1,*], David Gomez-Zepeda [2, 3], Arthur Declercq [4, 5], Mateusz K. Łącki [2], Seymen Avci [1], Konstantin Bob [1], Ute Distler,[2, 3], Thomas Michna[2, 3], Lennart Martens [4, 5], Stefan Tenzer [2, 3] and Andreas Hildebrandt [1]

[1] Institute of Computer Science, Johannes Gutenberg University, Mainz, 55128, Germany,
[2] Institute for Immunology, University Medical Center of the Johannes Gutenberg University, Mainz, 55128, Germany,
[3] Immunoproteomics Unit, Helmholtz-Institute for Translational Oncology (HI-TRON), Mainz, 55131, Germany,
[4] VIB-UGent Center for Medical Biotechnology, VIB, 9052, Gent, Belgium,
[5] Department of Biomolecular Medicine, Ghent University, 9000, Ghent, Belgium.

*To whom correspondence should be addressed.

Abstract

1 Supplementary Material and Methods

1.1 Summary

The methodologies for sample preparation and LC-MS analysis are summarized in this paragraph and detailed in the following sections. All reagents used were analytical or LC-MS grade, and LoBind tubes (Eppendorf) were employed to minimize sample loss. Previously published protocols were employed, as referenced in the following lines. Briefly, whole-cell lysates were obtained from HeLa, *E. coli*, yeast (*Saccharomyces cerevisiae bayanus*), or mouse brain. Samples were FASP-digested using trypsin Sielaff *et al.* (2017); Wisniewski *et al.* (2009). The digests were utilized for direct injections (HeLa), mixed into hybrid-proteome standards composed of HeLa, yeast, *E. coli* (HYE) Navarro *et al.* (2016) at ratios of 65% wt/wt human, 30% wt/wt yeast and 5% wt/wt *E. coli* (HYEA) or 65% wt/wt human, 15% wt/wt yeast and 20% wt/wt *E. coli* (HYEB), or employed for phosphopeptide enrichment by TiOtextsubscript2 (GL Sciences, Tokyo, Japan). Immunopeptidomics samples were prepared from a non-denaturant lysate of $5x10^8$ JY cells (1.2% CHAPS in PBS) or 5 mL of clarified commercial plasma. MHC/HLA class I ligands were enriched using an anti panHLA-(A, B, C) antibody W6/32 purchased from Hölzel Biotech (Cologne, Germany) and produced by Leinco Technologies (St. Louis, Missouri, USA). The MHC ligands were then eluted in acidic conditions (0.2% TFA), clarified by ultrafiltration (10 kDa MWCO), and desalted by SPE in Oasis HLB 96-well plates (Waters) Hahlbrock (2017). All the samples were dried in a vacuum centrifuge at the last stage and dissolved in 0.1% formic acid (FA) in MS-grade water for LC-MS analysis. Diverse LC-MS platforms and methods were employed, depending on the sample. In summary, timsTOF Pro-2 or timsTOF SCP Brunner *et al.* (2022) (Bruker) MS were used, connected to either nanoAcquity (Waters) or nanoElute (Bruker) chromatography systems. The peptide samples were directly injected and separated in Aurora-series C18 analytical columns (IonOpticks). MS data was acquired in DDA-PASEF Meier *et al.* (2018).

1.2 Biological samples

Cell and yeast culture. The human cervix carcinoma cell line HeLa was obtained from the German Resource Centre for Biological Material (DSMZ). Cells were cultured in Iscove's Modified Dulbecco's Medium (IMDM; PAN Biotech, Aidenbach, Germany) supplemented with 10% (v/v) fetal calf serum (FCS; Thermo Fisher Scientific (Invitrogen), Waltham, MA), 1% (v/v) L-glutamine (Carl Roth), and 1% (v/v) sodium pyruvate (Serva, Heidelberg, Germany) at 37°C in a 5% CO_2 environment and harvested at 70% confluence. Cells were washed once with phosphate-buffered saline (PBS; Carl Roth) and detached from the culture flasks with 0.05% Trypsin-EDTA solution (Sigma-Aldrich). The human B lymphoblastoid cell line JY was purchased from ATCC. JY cells were grown suspension in RPMI1640 medium supplemented with 10 % FCS (Gibco), 2 mM glutamine, 1 mM sodium pyruvate, 100 units/ml penicillin, and 100 µg/ml streptomycin. Saccharomyces cerevisiae bayanus, strain Lalvin EC-1118 was obtained from the Institut Oenologique de Champagne and grown in YPD medium. Harvested HeLa, JY, and yeast cells were transferred into centrifugal tubes and washed three times with PBS before being frozen and stored at -80°C until further processing.

Animal tissue. Mice (C57BL/6) were sacrificed by CO2 asphyxiation. After decapitation, the brain was dissected, immediately frozen using liquid nitrogen, and stored at -80°C. The animal experiments were

© The Author 2015. Published by Oxford University Press. All rights reserved. For permissions, please e-mail: journals.permissions@oup.com

picture(0,0)(-35,0)(1,0)30 (0,-35)(0,1)30 picture picture(0,0)(35,0)(-1,0)30 (0,-35)(0,1)30 picture

conducted in accordance with national laws and approved by the local authorities.

Commercial human plasma and E. coli samples. Human blood plasma was purchased from XXY, aliquoted, and stored at -80°C until further processing. A tryptic protein digest of E. coli proteins (MassPREP standard) was purchased from Waters.

1.3 Sample preparation

Protein extraction. HeLa cells were lysed using a urea-based lysis buffer (7 M urea, 2 M thiourea, 5 mM dithiothreitol (DTT), 2% (w/v) CHAPS). JY cell pellets were thawed and lysed in 1% CHAPS in PBS. Tissue was ground in liquid nitrogen using a mortar and pestle. Proteins were extracted from the tissue powder adding a urea-based lysis buffer (8 M urea, 2 M thiourea in 100 mM NH4HCO3, pH 7.4). Lysis was further promoted by sonication at 4°C for 15 min (30 s on/30 s off) using a Bioruptor device (Diagenode, Liège, Belgium). After cell lysis, protein concentration was determined using the Pierce 660 nm (for HeLa) or Pierce BCA protein assays (for JY, due to the CHAPS) according to the manufacturer´s protocols (Thermo Fisher Scientific).

Protein digestion for whole-proteome samples HeLa, JY, Yeast, and plasma samples were processed using filter-aided sample preparation (FASP) as detailed before (Wisniewski *et al.* (2009), Sielaff *et al.* (2017)). In brief, lysates were loaded onto spin filter columns (Nanosep centrifugal devices with Omega membrane, 30 kDa MWCO; Pall, Port Washington, NY) and washed three times with buffer containing 8 M urea. Afterward, proteins were reduced and alkylated using DTT and iodoacetamide (IAA), respectively. After alkylation, excess IAA was quenched by the addition of DTT. Then, the buffer was exchanged by washing the membrane three times with 50 mM NH4HCO3. The proteins were digested overnight at 37°C using trypsin (Trypsin Gold, Promega, Madison, WI) at an enzyme-to-protein ratio of 1:50 (w/w). After proteolytic digestion, peptides were recovered by centrifugation and two additional washes with 50 mM NH4HCO3. After combining peptide flow-throughs, samples were acidified with trifluoroacetic acid (TFA) to a final concentration of 1% (v/v) trifluoroacetic acid (TFA) and lyophilized. Lyophilized peptides were reconstituted in 0.1% (v/v) formic acid (FA) for LC-MS analysis.

Preparation of phosphopeptide samples. After reduction and alkylation with DTT and IAA, proteins were digested in-solution overnight at 32 °C using trypsin (Pierce TPCK-Trypsin, Thermo Scientific) at an enzyme-to-protein ratio of 1:25 (w/w). After overnight digestion, peptides were desalted using SepPak tC18 100 mg cartridges (Waters Corporation) and lyophilized. Phosphopeptide enrichment was performed using preloaded TiO_2 spin-tips (3 mg TiO_2, 200 µL tips, GL Sciences, Tokyo, Japan). The tips were conditioned at room temperature (RT) by centrifugation (3,000 xg, 2 min) passing through 20 µL of wash buffer (80 % (v/v) acetonitrile (ACN), 0.4 % (v/v) TFA) followed by 20 µL of loading buffer (57 % (v/v) ACN, 0.3 % (v/v) TFA, 40 % (v/v) lactic acid) applying the same settings for centrifugation. The peptides (1 mg per sample) were resuspended in 150 µL loading buffer, loaded onto the spin-tips, and centrifuged (1,000 xg, 10 min, RT). The flow-through was re-applied and centrifuged with the same settings. Bound phosphopeptides were first washed with 20 µL loading buffer followed by three wash steps with 20 µL wash buffer (all at 3,000 xg, 2 min, RT). Purified phosphopeptides were then eluted by centrifugation (1,000 xg, 10 min, RT), adding first 50 µL of 1.5 % (v/v) NH4OH followed by 50 µL of 5 % (v/v) pyrrolidine. Eluted phosphopeptides were acidified by adding 100 µL 2.5 % (v/v) TFA and desalted using Pierce graphite spin-columns (Thermo Scientific) following the manufacturer's protocol. After elution and lyophilization, the phosphopeptides were reconstituted in 20 µL 0.1 % (v/v) formic acid (FA) for LC–MS analysis.

Preparation of MHC ligand immunopeptidomics samples. JY cell lysate (in 1% (m/v) CHAPS in PBS) or undiluted plasma were used for MHC class I ligand enrichment. Immunoprecipitation was performed using an anti-panHLA Class I antibody (W6/32, purchased from Hölzel Biotech, Cologne, Germany) immobilized on CNBr-activated beads. The lysate was incubated with the Antibody-beads overnight, washed once with PBS and once with H_2O. Then, peptide ligands were eluted with 0.2% (v/v) TFA before being ultrafiltered (10 kDa cutoff) and then desalted by SPE on a Hydrophilic-Lipophilic-Balanced sorbent (HLB, Waters Corp.), using 35% (v/v) ACN + 0.1% (v/v) TFA for elution (Hahlbrock (2017)). Finally, dried peptides were reconstituted in 0.1% (v/v) FA for LC-MS/MS analyses.

1.4 Liquid-chromatography mass spectrometry (LC-MS)

Liquid-chromatography mass spectrometry (LC-MS). Reconstituted peptides were directly injected and separated on a nanoElute LC system (Bruker Corporation, Billerica, MA, USA) at 400 nL/min using a reversed-phase C18 column (Aurora 25 cm x 75 µm 1.6 µm, IonOpticks) attached to a MS. Eluting peptides were ionized in a CaptiveSpray Source (Bruker Corporation) and analyzed in positive mode ESI-MS on a timsTOF Pro 2 mass spectrometer Meier *et al.* (2018). Phosphopeptides were additionally analyzed in a timsTOF SCP mass spectrometer Brunner *et al.* (2022) (Bruker). Data were acquired using parallel accumulation serial fragmentation (PASEF) enhanced data-dependent (DDA) (Meier *et al.* (2018)).

nanoLC separation. The column was heated to 50°C. Mobile phase A was 0.1% FA (v/v) in water, and mobile phase B was 0.1% FA (v/v) in ACN. Peptides were loaded onto the column in direct injection mode at 600 bar and were separated, running a linear gradient from 2% to 37% mobile phase B over 38 min. Afterward, the column was rinsed at 95% B resulting in a total method time of 47 min. For the analysis on the timsTOF SCP, phosphopeptide samples were analyzed using a C18 Aurora UHPLC emitter column (15 cm x 75 µm 1.6 µm, IonOpticks), which was heated to 50°C. Peptides were loaded onto the column in direct injection mode at 600 bar and separated, running a linear gradient from 2% to 37% mobile phase B over 39 min at a flow rate of 400 nL/min. Then, the column was rinsed for 5 min at 95% B.

Analysis on the timsTOF Pro 2 or timsTOF SCP. In the timsTOF Pro 2 (Bruker), the dual TIMS was operated at a fixed duty cycle close to 100% using equal accumulation and ramp times of 100 ms, each spanning a mobility range from $1/K0 = 0.6Vscm^{-2}to1.6Vscm^{-2}$. The DDA-PASEF mode comprised ten PASEF scans per topN acquisition cycle (Meier *et al.* (2018)). Singly charged precursors were excluded from fragmentation by their position in the m/z–ion mobility plane. The collision energy was ramped linearly as a function of the mobility from $59eVat1/K0 = 1.3Vscm^{-2}to20eVat1/K0 = 0.85Vscm^{-2}$. In the timsTOF SCP, the high sensitivity detection mode was activated.

1.5 Peptide and protein identification from LC-MS raw files

Peptide identification software. The DDA raw files were processed by MaxQuant version 2.0.3.0 (Sinitcyn *et al.* (2021), Cox and Mann (2008)) or PEAKS XPro version 10.6 (BSI, Canada). For identification, FDR was set to 1%. For rescoring, FDR was set to 100%, and decoy peptides were included in the exported reports.

Protein databases. Phosphopeptide samples were searched using a custom compiled database containing UniprotKB/Swissprot entries of either the mouse reference proteome (UniProtKB release 2022_04, 17,107 entries), *Homo sapiens* (UniProtKB release 2022_02), or a merged list of human, yeast and *E. coli* (UniProtKB) proteomes. All databases were supplemented with a list of common contaminants. Default decoy database generation was used in each software for FDR calculation.

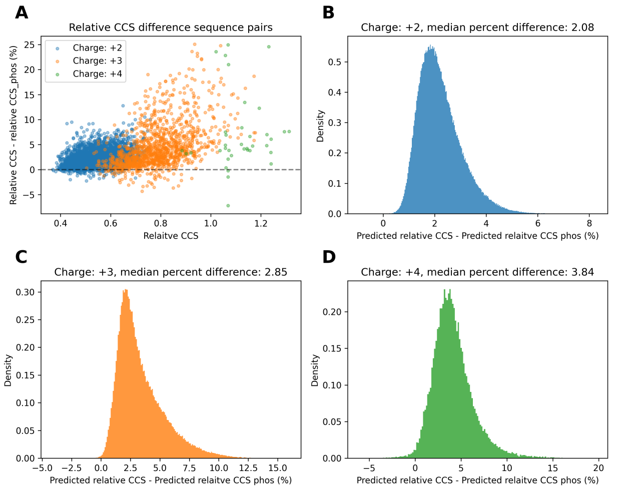

Fig. 1. Impact of phosphorylation on observed and predicted CCS values. A) Scatter plot showing pairwise experimentally observed differences between relative CCS of peptides with and without phosphorylation. X-axis represents relative CCS (CCS / m/z) of unphosphorylated peptides, Y-axis represents relative CCS of unphosphorylated peptides minus relative CCS of phosphorylated peptides in percent, charge states are color coded. B), C), D) Distribution of relative pairwise differences of predicted CCS values between peptides with and without phosphorylation for all modeled charge states. Phosphorylations were added to a set of sequences in-silico at random and CCS values where predicted for both versions (modified and unmodified) of a given sequence and charge. Since phosphorylation also increases peptide m/z, predicted CCS values are normalized by peptide m/z. It can be observed that for phosphorylated peptides, relaitve CCS values are decreased compared to the unmodified charge states. Difference is calculated by dividing CCS values by m/z and then subtracting resulting values of phosphorylated peptides from unphosphorylated peptides.

Whole proteome data processing in MaxQuant. Trypsin was set as digestion enzyme, allowing up to two missed cleavages and three modifications per peptide. Carbamidomethylation at cysteines was set as a fixed modification. Methionine oxidation and N-term acetylation were set as variable modifications. Peptides were identified with resolution thresholds of 15 ppm for MS1 and 0.01 Da for MS2.

Phosphopeptide data processing in PEAKS. Trypsin was set as digestion enzyme, allowing up to two missed cleavages. Carbamidomethylation at cysteines was set as fixed modification. Methionine oxidation, N-term acetylation as well as phosphorylation on serine, threonine and tyrosine were set as variable modifications allowing a maximum of five variable modifications per peptide. Peptides were identified with resolution thresholds of 15 ppm for MS1 and 0.03 Da for MS2. In addition to the 1% FDR threshold, PTMs were filtered during data post-processing to conserve only identifications with a a PTM AScore above 20 for a theoretical confidence of 99% of the PTM location.

MHC ligand data processing using PEAKS. Protein *in silico* digestion was configured to unspecific cleavage and no enzyme. Methionine oxidation, cysteine cysteinylation, and Protein N-ter acetylation were all configured as variable modifications with a maximum of two modifications per peptide. Peptides were identified with resolution thresholds of 15 ppm for MS1 and 0.03 Da for MS2.

2 Supplementary Figures

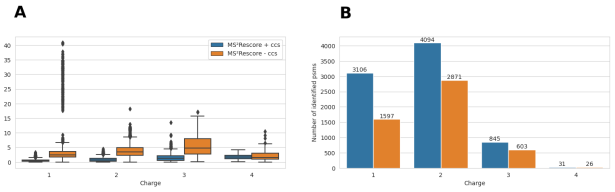

Fig. 2. Peptide spectrum matches resulting from rescoring with and without CCS features. A) Boxplots showing the percentual CCS error by charge for identifications exlusively identified with and without CCS errors as features during rescoring. B) Barplot showing number of PSMs by charge.

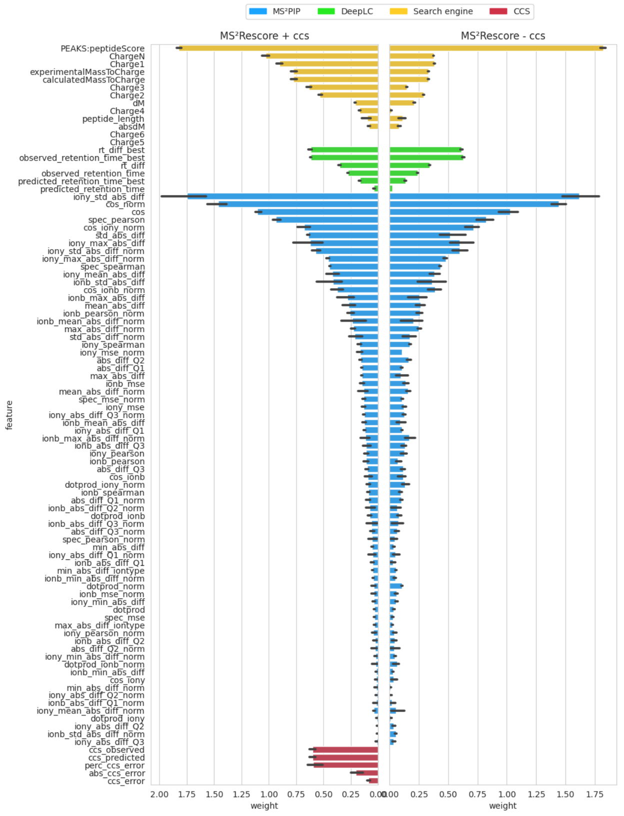

Fig. 3. Distribution of absolute normalized percolator feature weights for the different features used in MS²Rescore with CCS (left) and without CCS features (right) for the rescoring of the sinlgy charged MHC ligands with standard deviations for the different cross validations.

References

Brunner, A., Thielert, M., Vasilopoulou, C., Ammar, C., Coscia, F., Mund, A., Hoerning, O. B., Bache, N., Apalategui, A., Lubeck, M., Richter, S., Fischer, D. S., Raether, O., Park, M. A., Meier, F., Theis, F. J., and Mann, M. (2022). Ultra-high sensitivity mass spectrometry quantifies single-cell proteome changes upon perturbation. *Molecular Systems Biology*, **18**(3), 1–15.

Bush, M. F., Campuzano, I. D. G., and Robinson, C. V. (2012). Ion Mobility Mass Spectrometry of Peptide Ions: Effects of Drift Gas and Calibration Strategies. *Analytical Chemistry*, **84**(16), 7124–7130. Publisher: American Chemical Society.

Cox, J. and Mann, M. (2008). MaxQuant enables high peptide identification rates, individualized p.p.b.-range mass accuracies and proteome-wide protein quantification. *Nature Biotechnology*, **26**(12), 1367–1372.

Feola, S., Chiaro, J., Martins, B., Russo, S., Fusciello, M., Ylösmäki, E., Bonini, C., Ruggiero, E., Hamdan, F., Feodoroff, M., Antignani, G., Viitala, T., Pesonen, S., Grönholm, M., Branca, R. M., Lehtiö, J., and Cerullo, V. (2022). A novel immunopeptidomic-based pipeline for the generation of personalized oncolytic cancer vaccines. *eLife*, **11**, e71156. Publisher: eLife Sciences Publications, Ltd.

Hahlbrock, J. (2017). *MHC-Klasse-I vermittelte Antigenpräsentation : systembiologische Analyse in humanen Krebszelllinien und Charakterisierung der ER-residenten Aminopeptidase ERMP1*. Ph.D. thesis, Johannes Gutenberg-Universität Mainz.

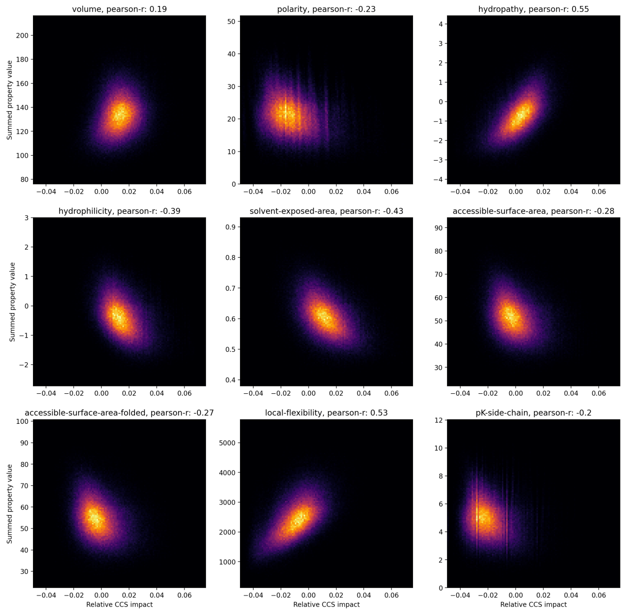

Fig. 4. Linear correlation between relative deep contribution of sequences and additive sequence property descriptors. Most impact was observed for hydropathy and local flexibility on relative CCS. All calculated pearson correlations calculated for relative increase or decrease in CCS with respect to the inital fit baseline.

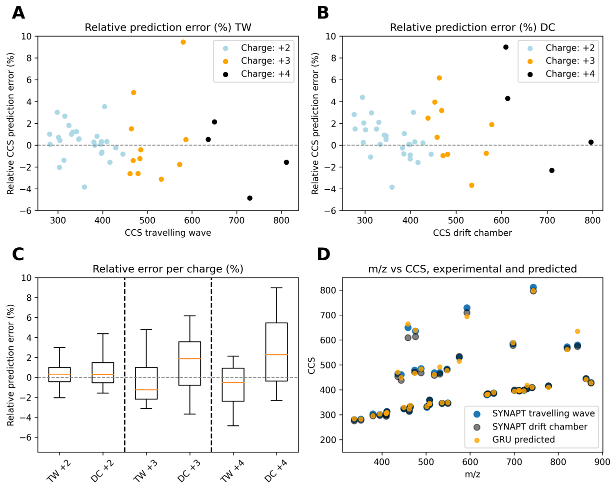

Fig. 5. Predicted vs observed CCS values of `ionmob` GRU predictor compared to data generated from both a TWIMS and DTIMS device. A), B) Observed CCS values vs relative prediction error for TWIMS (left) and DTIMS (right), charge states are color coded. C) Boxplots showing charge state wise relative prediction error for both devices. D). The m/z vs CCS plane showing all datapoints, measured with TWIMS, DTIMS and in-silico predicted. Data was extracted from Bush et al. (2012).

Meier, F., Brunner, A.-D., Koch, S., Koch, H., Lubeck, M., Krause, M., Goedecke, N., Decker, J., Kosinski, T., Park, M. A., Bache, N., Hoerning, O., Cox, J., Räther, O., and Mann, M. (2018). Online Parallel Accumulation–Serial Fragmentation (PASEF) with a Novel Trapped Ion Mobility Mass Spectrometer *. *Molecular & Cellular Proteomics*, **17**(12), 2534–2545. Publisher: Elsevier.

Navarro, P., Kuharev, J., Gillet, L. C., Bernhardt, O. M., MacLean, B., Röst, H. L., Tate, S. A., Tsou, C.-c., Reiter, L., Distler, U., Rosenberger, G., Perez-Riverol, Y., Nesvizhskii, A. I., Aebersold, R., and Tenzer, S. (2016). A multicenter study benchmarks software tools for label-free proteome quantification. *Nature Biotechnology*, **34**(11), 1130–1136.

Ogata, K., Chang, C.-H., and Ishihama, Y. (2021). Effect of phosphorylation on the collision cross sections of peptide ions in ion mobility spectrometry. *Mass Spectrometry*, **10**, 1–8.

Sielaff, M., Kuharev, J., Bohn, T., Hahlbrock, J., Bopp, T., Tenzer, S., and Distler, U. (2017). Evaluation of FASP, SP3, and iST Protocols for Proteomic Sample Preparation in the Low Microgram Range. *Journal of Proteome Research*, **16**(11), 4060–4072.

Sinitcyn, P., Hamzeiy, H., Salinas Soto, F., Itzhak, D., McCarthy, F., Wichmann, C., Steger, M., Ohmayer, U., Distler, U., Kaspar-Schoenefeld, S., Prianichnikov, N., Yılmaz, , Rudolph, J. D., Tenzer, S., Perez-Riverol, Y., Nagaraj, N., Humphrey,

picture(0,0)(-35,0)(1,0)30 (0,35)(0,-1)30 picture

picture(0,0)(35,0)(-1,0)30 (0,35)(0,-1)30 picture

S. J., and Cox, J. (2021). MaxDIA enables library-based and library-free data-independent acquisition proteomics. *Nature Biotechnology*, **39**(12), 1563–1573.

Wisniewski, J. R., Zougman, A., Nagaraj, N., and Mann, M. (2009). Universal sample preparation method for proteome analysis. *Nat Methods*, **6**(5), 359–362.

picture(0,0)(-35,0)(1,0)30 (0,-35)(0,1)30 picture

picture(0,0)(35,0)(-1,0)30 (0,-35)(0,1)30 picture

“output” — 2023/6/29 — page 5 — #5

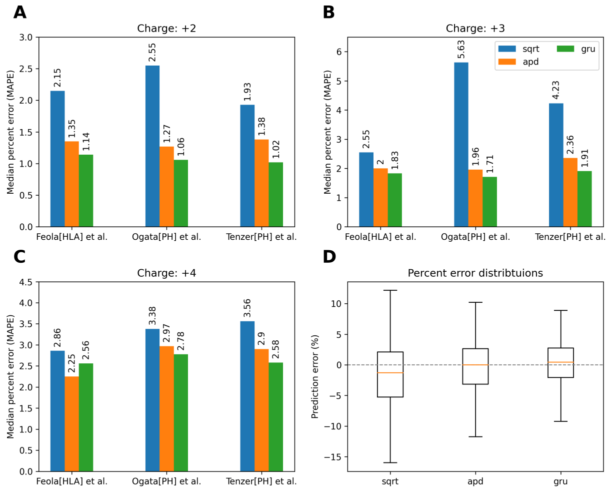

Fig. 6. Model performance comparison between baseline square-root fit, `AlphaPeptDeep` model and `ionmob` GRU predictor. A), B), C) Performance is shown per charge state on three different test datasets, containing MHC Feola et al. (2022) and phopsphorylated peptides Ogata et al. (2021). D) Boxplots showing relative error distributions of the three different models.

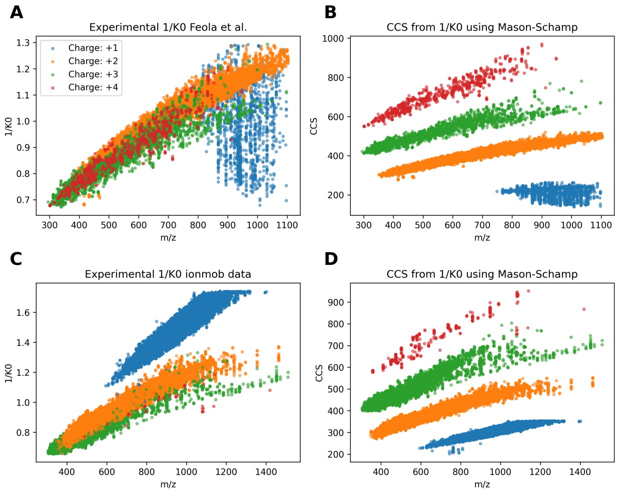

Fig. 7. Experimentally determined m/z vs inverse reduced mobility, $1/K0$, and translation of $1/K0$ to CCS using the Mason-Schamp equation. A), C) Experimentally measured $1/K0$ values for MHC peptides published by Feola et al. (2022) and a newly generated in-house MHC peptide dataset. It can be observed that the determined $1/K0$ values in A) are unreliable to accuratly calculate CCS values, see B). However, the altered device settings in C) allow for a informative translation to CCS in D).
